# Tolerability, pharmacokinetics, and pharmacodynamics of mirogabalin in healthy subjects: Results from phase 1 studies

**DOI:** 10.1002/prp2.418

**Published:** 2018-08-23

**Authors:** Karen Brown, Jeanne Mendell, Shoichi Ohwada, Ching Hsu, Ling He, Vance Warren, Victor Dishy, Hamim Zahir

**Affiliations:** ^1^ Daiichi Sankyo Pharma Development Basking Ridge New Jersey; ^2^ Daiichi Sankyo Co., Ltd. Tokyo Japan

**Keywords:** clinical pharmacology, clinical trial, drug‐food interactions, pharmacodynamics, pharmacokinetics and drug metabolism

## Abstract

Three phase 1 pharmacokinetic (PK)/pharmacodynamics (PD) studies were conducted in healthy men and women to further characterize the safety, tolerability, and PK/PD of mirogabalin administration with or without food and to guide the dose selection and regimen for phase 2 and 3 clinical development. The 3 studies included 2 randomized, double‐blind, placebo‐controlled, single‐ and multiple‐ascending‐dose studies, and 1 open‐label, crossover study to evaluate the PK of mirogabalin administered under fasting and fed (high‐fat meal) conditions. Forty‐eight and 47 healthy volunteers completed the single‐ and multiple‐dose studies, respectively. Thirty subjects were enrolled and completed the food effect study. Mirogabalin was well tolerated in the fed and fasted states. The most frequent treatment‐emergent adverse events (TEAEs)—dizziness and somnolence—were expected based on mirogabalin's mechanism of action. Subjects receiving the highest mirogabalin doses (50 and 75 mg single dose) showed greater dizziness and sedation and higher rates of TEAEs than subjects receiving 3‐30 mg. After oral administration, mirogabalin was rapidly absorbed (time to maximum concentration, ∼1 hour) and eliminated through urine unchanged (61%‐72% urinary excretion). Exposure increased in a dose‐proportional manner after single or multiple mirogabalin doses. No significant accumulation occurred with multiple doses over 14 days. After single doses of mirogabalin (15 mg), the bioavailability was considered equivalent in the fed and fasted states, indicating that mirogabalin can be taken without food restrictions. Based on these data, mirogabalin 15 mg twice daily was selected as the highest target dose for further clinical development.

**Abbreviation**
ADPS: average daily pain score
AE: adverse event
Ae: amount of parent drug or its metabolites excreted in urine
Ae0‐72: amount of parent drug or its metabolites excreted in urine over the 72‐h collection interval
AUC0‐12: area under the plasma concentration‐time curve from time 0–12 h
AUC0‐τ: area under the plasma concentration‐time curve for dosing interval
AUCinf: area under the plasma concentration‐time curve from time 0 extrapolated to infinity
BARS: brief ataxia rating scale
CI: confidence interval
CL/F: apparent total body clearance
CLr ss: renal clearance (at steady state)
CLss/F: apparent total body clearance after oral administration (at steady state)
*C*_max_: maximum observed concentration in plasma
*C*_max_ ss: maximum observed concentration in plasma (at steady state)
CNS: central nervous system
C‐SSRS: Columbia suicide severity rating scale
CV: coefficient of variation
DPNP: diabetic peripheral neuropathic pain
DSST: Digit Symbol Substitution Test
Fe: cumulative fraction excreted unchanged parent in urine
Fe0‐72: cumulative fraction of the dose excreted as unchanged parent in urine over the 72‐h collection interval
Fe0‐τ: cumulative fraction of the dose excreted as unchanged parent in urine over the entire collection interval
LARS: Line analog rating scale
LC‐MS/MS: liquid chromatography‐tandem mass spectrometry
LSM: least‐squares mean
MedDRA: Medical Dictionary for Regulatory Activities
PK: pharmacokinetic
PD: pharmacodynamics
Robs: observed accumulation ratio
*t*_1/2_: half‐life
TEAE: treatment‐emergent adverse event
*T*_max_: time to maximum observed concentration
VSS‐SF: vertigo symptom scale short form
Vz/F: apparent volume of distribution

## 1 INTRODUCTION

The α_2_δ‐1 subunit of Cav1‐ and Cav2‐type voltage‐gated calcium channels plays a role in neuropathic pain.^1^, ^2^, ^3^ Ligands of the α_2_δ‐1 subunit reduce Ca^2+^ influx into central nervous system (CNS) neurons and exert analgesic effects.^2^, ^3^, ^4^ As such, this subunit is the primary therapeutic target for 2 marketed neuropathic pain treatments; pregabalin and gabapentin.^5^, ^6^ Mirogabalin monobenzenesulfonate (Daiichi Sankyo Co., Ltd., Tokyo, Japan, herein referred to as mirogabalin) is a preferentially selective ligand of the α_2_δ‐1 subunit in development for treatment of neuropathic pain.^1^, ^2^, ^3^ In preclinical studies, mirogabalin demonstrated sustained analgesic effects in animal models of pain.^1^ Mirogabalin also demonstrated improved analgesia with a wider safety margin than pregabalin.^1^ In a phase 2 U.S. study of patients with diabetic peripheral neuropathic pain (DPNP; *n* = 452), average daily pain scores (ADPSs) were significantly reduced by mirogabalin 15, 20, and 30 mg/day compared with placebo after 5 weeks’ treatment. Mirogabalin administered at 30 mg/day (15 mg twice daily [BID]) met the criteria of minimally meaningful effect (defined as a ≥1.0‐point decrease in ADPS compared with placebo).^7^

Phase 1 randomized studies in healthy adults were conducted to characterize initial safety, tolerability, pharmacokinetic (PK), and pharmacodynamic (PD) profiles of mirogabalin and further characterize the effect of food on mirogabalin PK. These studies included double‐blind, placebo‐controlled, single‐ and multiple‐ascending‐dose studies and an open‐label, crossover study to evaluate the effects of mirogabalin administration with or without food. Various pharmacodynamic assessments were used to measure cognitive or nervous system‐related effects. Results of these studies guided the dosing regimen selected for phase 2 and 3 clinical development.

## 2 MATERIALS AND METHODS

### 2.1 Study design and subject selection

All 3 study protocols were reviewed and approved by the appropriate local independent institutional review board (INTEG REVIEW, Austin, TX, USA) and conducted in compliance with ethical principles originating from the Declaration of Helsinki and the International Conference on Harmonisation consolidated Guideline.

#### 2.1.1 Single‐ascending‐dose study

The single‐ascending‐dose study was a randomized, double‐blind, placebo‐controlled, 6‐cohort, sequential, escalating‐dose study to determine safety, tolerability, and PK parameters of mirogabalin in healthy subjects, conducted October 2010 to December 2010. This study enrolled healthy adults aged 18‐45 years with a body mass index (BMI) 19.0‐30.0 kg/m^2^. Detailed inclusion and exclusion criteria for all studies are reported in Data S1.

All subjects provided written informed consent before performing study‐specific evaluations. Six cohorts of subjects were dosed sequentially. Within each cohort, subjects were randomly assigned (6:2) to receive single oral doses of mirogabalin (3, 5, 10, 30, 50, or 75 mg) or placebo. Mirogabalin was provided as reconstituted powder (for the 3‐mg dose) and 5‐, 10‐, and 25‐mg tablets. Placebo was provided as matching reconstituted powder or tablets. A 7‐day minimum safety review period occurred between successive cohorts.

#### 2.1.2 Multiple‐ascending‐dose study

The multiple‐ascending‐dose study was a randomized, double‐blind, double‐dummy, placebo‐controlled, 5‐cohort, sequential, escalating‐dose study with pregabalin as an active control conducted to determine safety, tolerability, and PK parameters of mirogabalin in healthy elderly subjects, conducted January 2011 to April 2011. This study enrolled healthy adults aged 55‐75 years with a BMI 19.0‐32.0kg/m^2^. Five cohorts of subjects were dosed sequentially. Within each cohort, subjects were randomly assigned (6:2:2) to receive an oral dose of mirogabalin 5, 10, 15, 20, or 25 mg; pregabalin 150 mg; or placebo. All doses were given twice daily for 14 days except for mirogabalin 25 mg, which was given once daily for 5 days, twice daily for 8 days, and then 1 dose of 25 mg on the last day; and pregabalin, which was given 75 mg for 5 days, then 150 mg twice daily for 9 days. Mirogabalin was provided as 5‐ and 10‐mg tablets; pregabalin was provided as over‐encapsulated formulations at 75‐ and 150‐mg doses; placebo for mirogabalin and pregabalin was provided as matching tablets or capsules, respectively. A cohort could begin dosing as soon as the previous cohort completed day 14, if the safety profile of the previous cohort was acceptable. The effects of mirogabalin on PD parameters including sedation, attention, dizziness, and ataxia were also assessed.

#### 2.1.3 Food effect study

The food effect study was an open‐label, randomized, 2‐treatment, 2‐period, 2‐sequence crossover study conducted in healthy subjects to evaluate the PK of mirogabalin under fed vs fasted conditions, conducted December 2013. This study enrolled healthy adults aged 18‐60 years with a BMI 18.0‐30.0 kg/m^2^. A single oral dose of mirogabalin 15 mg was administered in 2 regimens, (A) fasted (overnight fast for ≥10 hours, followed by mirogabalin dosing and an additional 4‐hour fast) and (B) fed (overnight fast for ≥10 hours, followed by consumption of a high‐fat breakfast within 30 minutes and subsequent dosing of mirogabalin). Subjects were randomly assigned to treatments in the sequence AB or BA, with a ≥3‐day washout period between each treatment. Pharmacodynamic parameters, which included sedation, attention, dizziness, and ataxia, were also assessed.

### 2.2 Safety

For each of the 3 studies, the safety and tolerability was assessed for all subjects who received at least 1 dose of study medication. Assessment of safety was based on treatment‐emergent adverse events (TEAEs), clinical laboratory evaluations, vital signs, physical examinations, and electrocardiography. Adverse events (AEs) were coded using the latest version of the Medical Dictionary for Regulatory Activities (MedDRA) at the time of database lock (version 13.0 for the single‐ and multiple‐ascending‐dose studies, and version 15.1 for the food effect study).

In the multiple‐ascending‐dose and food effect studies, the Columbia suicide severity rating scale (C‐SSRS)^8^ was used to monitor suicidality. The C‐SSRS captures the occurrence, severity, and frequency of suicide‐related thoughts and behaviors, and was conducted by appropriately trained site personnel. Referral to a psychiatrist was to be made if the C‐SSRS showed significant findings.

### 2.3 Pharmacokinetic assessments

In the single‐ascending‐dose study, blood samples were taken before the mirogabalin dose and at 0.25, 0.5, 1, 1.5, 2, 2.5, 3, 4, 6, 8, 12, 24, 36, 48, 60, and 72 hours after dosing. Urine samples were collected within 2 hours before dosing and during the intervals 0‐4 hours, 4‐8 hours, 8‐12 hours, 12‐24 hours, 24‐36 hours, 36‐48 hours, and 48‐72 hours after dosing. Serial blood samples for the multiple‐ascending‐dose study were collected within 5 minutes before receiving the mirogabalin dose on study days 1, 3, 5, 8, 10, and 12; at 0.25, 0.5, 1, 1.5, 2, 2.5, 3, 4, 6, 8, and 12 hours after dosing on study days 1 and 14; and at 16, 24, 36, and 48 hours after dosing on study day 14. Serial urine samples were collected predose on study days 1 and 14; during the intervals 0‐4 hours, 4‐8 hours, 8‐12 hours, and 12‐24 hours after dosing on study days 1 and 14; and 24‐36 hours after dosing on study day 14. In the food effect study, blood samples were collected before the mirogabalin dose and at 0.5, 1, 1.5, 2, 2.5, 3, 3.5, 4, 6, 8, 10, 12, 14, 22, and 24 hours postdose.

Plasma concentrations of freebase mirogabalin were analyzed at Celerion (Lincoln, NE, USA) using a validated liquid chromatography‐tandem mass spectrometry method (LC‐MS/MS). The calibration curves for mirogabalin (1/*X* ^2^ weighting, linear regression) ranged from 1 to 1000 ng mL^−1^. For assay validation of mirogabalin, quality control samples were prepared at 1, 3, 75, 400, 750, and 1000 ng mL^−1^. Dilution integrity was verified at a concentration up to 20 000 ng mL^−1^. The intra‐ and interassay precision (coefficient of variation [CV]) values in validation were within 14.6% and 11.7%, respectively; the intra‐ and interassay accuracy values were −16.3% to 3.1%, and −7.1% to 1.9%, respectively.

Urine concentrations of freebase mirogabalin were analyzed at Celerion using a validated LC‐MS/MS method. The calibration curves for mirogabalin (1/*X* weighting, linear regression) ranged from 0.1 to 100 μg mL^−1^. In‐assay validation for mirogabalin, quality control samples were prepared at 0.100, 0.300, 7.50, 40.00, 75.0, and 100 μg mL^−1^. Dilution integrity was verified at a concentration up to 200 μg mL^−1^. The intra‐ and interassay precision (CV) values in validation were within 18.3% and 12.8%, respectively; the intra‐ and interassay accuracy values were −11.1% to 8.0%, and −3.5% to 2.5%, respectively.

The PK analysis set included all subjects who received a dose of mirogabalin and had sufficient plasma concentration data for mirogabalin to characterize the PK parameters. The PK parameters were calculated using Phoenix WinNonlin (version 4.0 [single‐ and multiple‐ascending‐dose studies] and version 6.3 [food study], Certara, Princeton, NJ, USA) and included (as appropriate) maximum observed concentration in plasma (*C*_max_); time of maximum observed concentration (*T*_max_); area under the plasma concentration‐time curve for a dosing interval (AUC_0‐τ_, multiple‐ascending‐dose study only); area under the plasma concentration‐time curve from time 0 to the last measurable concentration (AUC_last_, multiple‐ascending‐dose study only); area under the plasma concentration‐time curve from time 0 extrapolated to infinity (AUC_inf_); terminal half‐life (*t*_1/2_); apparent volume of distribution (*V*_z_/*F*); observed accumulation ratio (*R*_obs_), calculated as AUC_0‐τ_, (day 14)/area under the plasma concentration‐time curve from time 0 to 12 hours (AUC_0‐12_) (day 1); renal clearance; apparent total body clearance (CL/F); the amount of parent drug or its metabolites excreted in urine during each collection interval; and cumulative fraction of the dose excreted as unchanged parent in urine during each collection interval (Fe).

Mirogabalin plasma concentrations were summarized descriptively; plasma and urine concentration‐time data were analyzed by noncompartmental methods, with concentrations below the limit of quantitation set to 0. In the single‐ and multiple‐ascending‐dose studies, the relationship between dose and PK parameters was examined using a graphical approach and linear regression of dose‐normalized parameters. Apparent dose proportionality of PK parameters (single dose, AUC_inf_, AUC_last_, and *C*_max_; multiple dose, day 14 AUC_0‐τ_ and steady state *C*_max_) were assessed graphically and using a linear regression analysis of dose‐normalized parameters.

In the food effect study, peak and total mirogabalin exposures were compared between fasted (A) and fed (B) conditions using a mixed‐effects model for the log‐transformed PK values with treatment sequence, period and treatment as fixed effects, and subject nested with sequence fitted as a random effect. Geometric mean ratios of Treatment BA were calculated by exponentiation of the differences in least‐squares mean (LSM), along with corresponding 90% confidence intervals (CIs). An absence of food effect was determined if 90% CIs were entirely contained within the 80% to 125% equivalence interval.

### 2.4 Pharmacodynamic assessments

In the single‐ and multiple‐ascending‐dose studies, the PD analysis set included all subjects who received a dose of study medication and for whom at least 1 postdose PD assessment was available. PD variables were evaluated at each measurement point using descriptive statistics and graphics.

The PD variables in the single‐ and multiple‐ascending‐dose studies were selected to elucidate the CNS‐related tolerability profile of mirogabalin and evaluated sedation, attention, dizziness, and ataxia. More detail about each assessment is provided in Supplementary Data. No PD assessments were performed in the food effect study. Sedation was analyzed in the single‐ and multiple‐ascending‐dose studies using the Line Analog Rating Scale (LARS).^9^, ^10^ Attention was measured by the Digit Symbol Substitution Test (DSST)^11^. Dizziness was measured by the Vertigo Symptom Scale Short Form (VSS‐SF);^12^ and ataxia was measured by the Brief Ataxia Rating Scale (BARS).^13^ Full details of each scale are reported in Data S2.

In the single‐ascending‐dose study, all scales were assessed before dosing and at 2, 7, and 24 hours after dosing (in order: LARS, DSST, VSS‐SF, BARS); the BARS was additionally assessed at 12 hours after dosing. In the multiple‐ascending‐dose study, LARS, DSST, VSS‐SF, and BARS were assessed on study days −1, 1, 3, 6, 8, and 13, at 2 hours (except BARS) and 7 hours postdose (or the time matched hour on study day −1), or at early withdrawal.

## 3 RESULTS

### 3.1 Subject disposition and demographics

In the single‐ and multiple‐ascending‐dose studies, 48 healthy subjects were enrolled in each study. All 48 subjects in the single‐ascending‐dose study completed the study; 1 subject out of 48 from the multiple‐ascending‐dose study discontinued (on day 8 after 15 doses of study treatment) because of elevated hepatic transaminase levels.

Thirty subjects were enrolled in the food effect study and randomly assigned 1:1 to treatment sequence AB (*n* = 15) or BA (*n* = 15). All 30 subjects completed the study. Baseline demographics are reported in Table S1. More men than women enrolled in all 3 studies (45:3 in the single‐ascending‐dose study, 31:17 in the multiple‐ascending‐dose study, and 19:11 in the food effect study), and most subjects were White (56.3%, 91.7%, and 60.0%, respectively). Mean age was 31.4 years in the single‐ascending‐dose study and 35.9 years in the food effect study. In the multiple‐ascending‐dose study, which enrolled subjects aged 55‐75 years, mean age was 61.4 years.

### 3.2 Safety

In the single‐dose study, most TEAEs were reported in the 50‐ and 75‐mg dose cohorts (Table 1); lower doses (≤30 mg) were well tolerated. The most common TEAEs after mirogabalin dosing were somnolence (20.8%) and dizziness (18.8%). At doses higher than 30 mg, unsteady gait, nausea/vomiting, and blurred vision were observed and were dose limiting. In the multiple‐ascending‐dose study, doses of mirogabalin 5, 10, and 15 mg BID were well tolerated; however, the 15‐mg BID dose was associated with a higher incidence of TEAEs, most notably dizziness/somnolence (Table 1). Doses of mirogabalin 20 and 25 mg twice daily were not well tolerated. Moderate somnolence was reported in 1, 2, and 3 subjects in the mirogabalin 10‐, 20‐, and 25‐mg groups, respectively. Somnolence was mild in the 15‐mg group. Moderate dizziness was reported by 2 subjects in the 15‐mg group and 1 subject each in the pregabalin and mirogabalin 25‐mg group. Moderate cognitive disorder was reported by 3 and 1 subjects in the mirogabalin 20‐ and 25‐mg groups, respectively. One subject in the mirogabalin 20‐mg group reported moderate balance disorder. Moderate visual impairment was reported by 2 subjects in the 25‐mg group. CNS‐related TEAEs (somnolence, dizziness, balance disorder, cognitive disorder) resolved or improved within 4‐5 days of continued dosing. Single‐dose mirogabalin 15 mg was well tolerated when administered with or without food; TEAEs were reported in 5 subjects (palpitations [*n* = 2], headache [*n* = 1], somnolence [*n* = 1], and dysmenorrhea [*n* = 1]).

**Table 1:** Treatment‐emergent adverse events

Single‐ascending‐dose study								
TEAE, n (%)	Mirogabalin						Placebo (*n* = 12)	Overall (*N* = 48)
	3 mg (*n* = 6)	5 mg (*n* = 6)	10 mg (*n* = 6)	30 mg (*n* = 6)	50 mg (*n* = 6)	75 mg (*n* = 6)		
Any TEAE	2 (33.3)	2 (33.3)	3 (50.0)	2 (33.3)	6 (100.0)	6 (100.0)	6 (50.0)	27 (56.3)
Most common TEAEs (≥2 subjects in any dose cohort)								
Somnolence	0	1 (16.7)	0	1 (16.7)	4 (66.7)	3 (50.0)	1 (8.3)	10 (20.8)
Dizziness	0	0	1 (16.7)	1 (16.7)	3 (50.0)	4 (66.7)	0	9 (18.8)
Nausea	1 (16.7)	0	0	1 (16.7)	2 (33.3)	2 (33.3)	0	6 (12.5)
Headache	1 (16.7)	0	1 (16.7)	0	0	4 (66.7)	0	6 (12.5)
Vision blurred	0	0	0	0	0	3 (50.0)	0	3 (6.3)
Tremor	0	0	0	0	0	2 (33.3)	0	2 (4.2)

Multiple‐ascending‐dose study								
TEAE, n (%)	Mirogabalin					Pregabalin 150 mg BID (*n* = 8)	Placebo (*n* = 10)	Overall (*N* = 48)
	5 mg BID (*n* = 6)	10 mg BID (*n* = 6)^a^	15 mg BID (*n* = 6)	20 mg BID (*n* = 6)	25 mg QD to BID (*n* = 6)			
Any TEAE	3 (50.0)	5 (83.3)	6 (100.0)	5 (83.3)	6 (100.0)	5 (62.5)	4 (40.0)	34 (70.8)
Most common TEAEs (≥2 subjects in any dose cohort)								
Somnolence	1 (16.7)	2 (33.3)	3 (50.0)	4 (66.7)	4 (66.7)	1 (12.5)	0	15 (31.3)
Constipation	0	0	3 (50.0)	2 (33.3)	5 (83.3)	1 (12.5)	2 (20.0)	13 (27.1)
Headache	2 (33.3)	1 (16.7)	3 (50.0)	1 (16.7)	1 (16.7)	2 (25.0)	0	10 (20.8)
Dizziness	0	0	5 (83.3)	1 (16.7)	2 (33.3)	1 (12.5)	0	9 (18.8)
Balance disorder	0	0	1 (16.7)	3 (50.0)	0	1 (12.5)	0	5 (10.4)
Cognitive disorder	0	0	0	3 (50.0)	1 (16.7)	0	0	4 (8.3)
Visual impairment	0	0	1 (16.7)	1 (16.7)	2 (33.3)	0	0	4 (8.3)
Insomnia	0	0	2 (33.3)	0	0	1 (12.5)	0	3 (6.3)
Flatulence	0	0	0	0	2 (33.3)	1 (12.5)	0	3 (6.3)
Vision blurred	0	0	0	2 (33.3)	0	0	0	2 (4.2)
Disturbance in attention	0	0	2 (33.3)	0	0	0	0	2 (4.2)
Gait disturbance	0	0	0	0	2 (33.3)	0	0	2 (4.2)

BID, twice daily; QD, once daily; TEAE, treatment‐emergent adverse event.

a The single subject who discontinued the study early had a mild treatment‐emergent adverse event of elevated hepatic transaminase level.

No deaths or serious AEs were reported in any of the studies, and only 1 subject withdrew because of a TEAE; a 64‐year‐old woman in the multiple‐ascending‐dose study receiving mirogabalin 10 mg BID. She had asymptomatic elevated hepatic transaminase levels (aspartate aminotransferase/alanine transaminase up to 4.1/4.3 times the upper limit of normal without increased bilirubin level) that started on day 8. The subject withdrew from the study on day 8 and the TEAE resolved 16 days later. Most TEAEs were mild or moderate; however, in the single‐ascending‐dose study, 2 subjects receiving 75 mg mirogabalin reported multiple severe TEAEs and the subjects were significantly impaired for up to 3 days postdose. TEAEs included dizziness, somnolence, and unsteady gait (all deemed severe); nausea/vomiting and blurred vision (both deemed moderate); and tremulousness and headache (both deemed mild); all resolved without treatment. In 1 of these subjects, a fixed drug eruption (ie, an allergic reaction to medication recurring at the same site) on the right cheek was noted on day 3. A topical corticosteroid was used for 12 days and the TEAE was still present at the end of the study.

A trend toward increased standing blood pressure was also observed in the 50‐ and 75‐mg dose cohorts; however, this was driven by a small number of subjects with large asymptomatic changes at each dose. Therefore, the clinical relevance of this observation is unclear. No other findings of clinical significance were observed in clinical laboratory evaluations, vital sign assessment, electrocardiography, physical examination, or results of the C‐SSRS in any of the studies.

### 3.3 Pharmacokinetics

#### 3.3.1 Single‐ascending‐dose study

**Figure 1:**
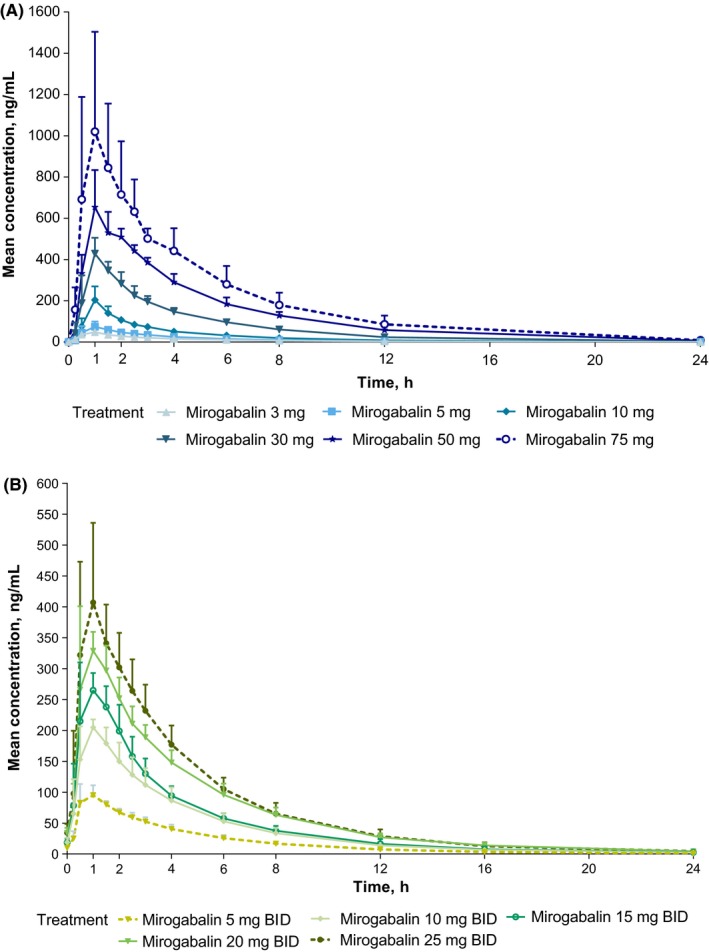
Mean concentration‐time profiles after administration of mirogabalin. Beyond 24 hours, the plasma concentration remained at 0 for all treatment arms in both studies and is not shown. (A) Single‐ascending‐dose study: mirogabalin 3‐75 mg on study day 1. (B) Multiple‐ascending‐dose study: mirogabalin 5‐25 mg BID on study day 14. BID, twice daily

Mean mirogabalin plasma concentrations increased in a dose‐proportional manner (Figure 1A), as did exposure (Table 2). Mirogabalin was rapidly absorbed; mean clearance and volume of distribution were similar across the doses. *T*_max_ occurred at 1 hour and mean half‐life ranged from 3.0 to 4.9 hours. Dose‐normalized values of *C*_max_ and AUC_inf_ were not significantly different across dose levels based on *P* values from the regression analysis (*P* > 0.05).

**Table 2:** Plasma and urinary pharmacokinetic parameters in the single‐ascending‐dose study

	Mirogabalin					
	3 mg (*n* = 6)	5 mg (*n* = 6)	10 mg (*n* = 6)	30 mg (*n* = 6)	50 mg (*n* = 6)	75 mg (*n* = 6)
Plasma parameters, study day 1						
*T*_max_, median (range), h	1.00 (0.50‐1.00)	1.00 (0.50‐2.00)	1.00 (1.00‐1.50)	1.00 (1.00‐1.50)	1.00 (1.00‐2.00)	1.00 (1.00‐1.50)
*C*_max_, ng/mL	49 (8.5)	78 (18.0)	205 (64.0)	433 (67.9)	671 (153.0)	1060 (459.0)
AUC_0‐inf_, ng·h/mL	184 (21.8)	276 (27.0)	614 (84.0)	1682 (233.4)	3231 (393.0)	4896 (1396)
*t*_1/2_, h	3.31 (0.37)	2.96 (0.17)	3.32 (0.75)	3.37 (0.26)	3.82 (0.32)	4.94 (2.93)
CL/F, L/h	16.50 (2.13)	18.24 (1.76)	16.55 (2.39)	18.09 (2.21)	15.67 (1.95)	16.19 (3.78)
*V*_z_/F, L	78.78 (13.89)	78.01 (8.64)	80.00 (27.20)	87.97 (13.31)	86.29 (12.99)	116.2 (75.76)
Urinary parameters, study day 1						
Ae_0‐72_, mg	1.90 (0.14)	3.42 (0.52)	7.15 (0.89)	20.43 (1.92)	32.52 (0.95)	45.57 (6.01)
Fe_0‐72_	0.63 (0.05)	0.68 (0.10)	0.71 (0.09)	0.68 (0.06)	0.65 (0.02)	0.61 (0.08)
CL_r_ L/h	10.41 (1.34)	12.39 (1.34)	11.74 (1.52)	12.39 (2.22)	10.19 (1.26)	9.96 (3.11)

Data are expressed as arithmetic mean (SD) unless otherwise specified.

Ae, the amount of parent drug or its metabolites excreted in urine during each collection interval; Ae_0‐72_, cumulative amount of drug excreted into the urine over the 72‐hour collection interval; AUC_0‐inf_, area under the plasma concentration‐time curve from the time of dosing extrapolated to infinity; CLr, renal clearance; *C*_max_, maximum observed concentration in plasma; Fe_0‐72_, cumulative fraction of the dose excreted as unchanged parent in urine over the 72‐hour collection interval; SD, standard deviation; *t*_1/2_, terminal half‐life; *T*_max_, time of maximum observed concentration (at steady state); V_z_/F, apparent volume of distribution, based on the terminal elimination phase.

Urinary PK data are shown in Table 2. Mirogabalin was rapidly eliminated via urinary excretion (61%‐72%), the majority during the first 0‐to‐4‐hour collection interval for all doses. The mean amount of mirogabalin excreted increased as the dose administered increased. The mean fraction of the dose excreted as unchanged mirogabalin was similar across dose levels.

#### 3.3.2 Multiple‐ascending‐dose study

Mean mirogabalin plasma concentrations increased with increasing doses (Figure 1B), although exposure seemed to increase in a slightly less than proportional manner. Plasma PK parameters for mirogabalin and pregabalin on day 14 are shown in Table 3. Mean clearance and volume of distribution were comparable across all mirogabalin dose levels. The mean half‐life of mirogabalin ranged from 3.6 to 7.5 hours, and *R*_obs_ was <1.5, indicating no significant plasma accumulation of mirogabalin on day 14. Steady state conditions were reached by day 3 for mirogabalin doses 5, 10, 15, and 20 mg BID and by day 8 (2 days from the first BID dosing on day 6, for mirogabalin 25 mg). Pregabalin PK parameters were comparable with those previously reported for healthy subjects.^14^

**Table 3:** Plasma and urinary pharmacokinetic parameters in the multiple‐ascending‐dose study

	Mirogabalin					Pregabalin150 mg BID (*n* = 8)
	5 mg BID (*n* = 6)	10 mg BID (*n* = 5)^a^	15 mg BID (*n* = 6)	20 mg BID (*n* = 6)	25 mg QD to BID (*n* = 6)	
Plasma parameters, study day 14						
*T*_max_, median (range), h	1.00 (0.50‐1.50)	1.00 (0.50‐1.00)	1.00 (0.50‐1.50)	1.00 (0.50‐1.50)	1.00 (0.50‐1.50)	1.27 (1.00‐2.50)
*C*_max ss_, ng/mL	97 (19.7)	211 (11.1)	296 (39.1)	354 (58.9)	426 (141.0)	6050 (1240.0)
AUC_0‐τ_, ng·h/mL	406 (48.5)	857 (141.9)	1033 (87.6)	1469 (168.2)	1710 (283.0)	40 360 (7818.0)
*t*_1/2_, h	3.58 (0.74)	4.55 (1.12)	4.23 (1.90)	5.80 (3.07)	7.49 (6.03)	7.06 (0.96)
CL_ss_/*F*, L/h	12.45 (1.37)	11.92 (1.87)	14.62 (1.35)	13.76 (1.55)	15.01 (2.87)	3.85 (0.81)
*V*_Z_/*F*_ss_, L	64.2 (15.8)	77.6 (19.5)	87.7 (34.5)	112.7 (52.1)	170.3 (153.8)	39.6 (11.9)
*R*_obs_	1.15 (0.10)	1.24 (0.11)	1.13 (0.20)	1.14 (0.09)	1.18 (0.10)	2.98 (0.42)
Urinary parameters, study day 14						
Ae_0‐τ_, mg	4.20 (1.20)	7.59 (3.21)	12.65 (2.46)	13.45 (4.81)	24.06 (4.52)	—
Fe_0‐T, ss_	0.84 (0.24)	0.76 (0.32)	0.84 (0.16)	0.67 (0.24)	0.96 (0.18)	—
CL_r ss_, L/h	10.34 (2.95)	8.81 (3.43)	12.29 (2.45)	9.52 (4.38)	14.20 (2.43)	—

Data are expressed as arithmetic mean (SD) unless otherwise specified.

Ae, the amount of parent drug or its metabolites excreted in urine during each collection interval; Ae_0‐τ_, cumulative amount of drug or metabolite excreted into the urine over the entire collection interval; AUC_0‐τ_, area under the plasma concentration‐time curve for a dosing interval; BID, twice daily; *C*_max ss_, maximum observed concentration in plasma (at steady state); CL_r ss_, renal clearance (at steady state); CL_ss_/*F*, apparent total body clearance after oral administration (at steady state); Fe_0‐T, ss_, cumulative fraction of the dose excreted as unchanged parent in urine over the entire collection interval (at steady state); QD, once daily; *R*_obs_, observed accumulation ratio, calculated as AUC_0‐τ_ (day 14)/AUC_0‐12_ (day 1); SD, standard deviation; *t*_1/2_, terminal half‐life; *T*_max_, time of maximum observed concentration (at steady state); *V*_z_/*F*_ss_, apparent volume of distribution (at steady state), based on the terminal elimination phase.

a One subject discontinued from the study early owing to a treatment‐emergent adverse event.

*C*_max_ and AUC_0‐τ_ values seemed to increase in a slightly less than proportional manner with increasing oral doses of mirogabalin. Dose‐normalized values of *C*_max_ were not significantly different across dose levels (*P *>* *0.05), whereas dose‐normalized values of AUC_0‐τ_ were significantly different across dose levels (*P *<* *0.05), based on the regression analysis.

Urinary PK data are shown in Table 3. The mean amount of mirogabalin excreted on day 14 increased in a dose‐proportional manner. The mean fraction of the dose excreted as unchanged mirogabalin over the 12‐hour collection interval (Fe_0‐12_) was similar across dose levels, ranging from 0.7 to 0.9. Mean renal clearance rates were similar across dose levels, ranging from 8.8 to 14.2 L/h.

#### 3.3.3 Food effect study

Although the mean total exposure was similar under fed and fasting conditions, the *C*_max_ for mirogabalin was reduced by approximately 18%; the geometric LSM ratio (90% CI) was 81.86 (75.33%‐88.95%), and T_max_ was delayed by 0.5 hours when administered under fed conditions, consistent with a food‐induced delay in gastric emptying.^14^ The geometric LSM (90% CI) of AUC_0‐inf_ was 94.16% (91.08%‐97.34%). Although exposure was 6% lower in fed vs fasted subjects, the 90% CIs were contained between 80% and 125%. The *t*_1/2_, *V*_z_/*F*, and CL/*F* of mirogabalin were unaffected by food.

### 3.4 Pharmacodynamics

#### 3.4.1 Sedation

Compared with those in other dose cohorts, subjects receiving higher doses of mirogabalin (30, 50, and 75 mg) in the single‐ dose study had greater levels of sedation at each postdose assessment, according to LARS (Figure S1A). For the 30‐ and 50‐mg cohorts, LARS scores returned to baseline by 24 hours postdose. Consistent with these findings, safety assessments indicated that somnolence was reported as a TEAE by more subjects in the 50‐mg cohort (66.7%) and the 75‐mg cohort (50%) relative to the lower dose cohorts (Table 1). By contrast, mirogabalin did not increase sedation in the multiple‐dose study (Figure S1B). Additionally, the profile of mood state was also used (data on file, methods in Supplemental Data) in both studies. The data for the profile of mood states had the same trend as LARS (data on file, Daiichi Sankyo, Inc., Basking Ridge, NJ), however, the frequency of somnolence reported as a TEAE seemed to increase as the mirogabalin dose increased (Table 1).

#### 3.4.2 Attention

Subjects in the highest dose cohorts (50 and 75 mg) had reduced attention compared with those in other dose cohorts, according to DSST (Figure S1C) in the single‐ascending‐dose study. However, disturbance in attention was reported as a TEAE by only 1 subject in the study (75‐mg cohort), suggesting that the detected deficits in attention were subtle and not clinically relevant. In the multiple‐dose study, attention was not decreased by any mirogabalin dose evaluated; rather, almost all DSST scores increased over time (Figure S1D). Disturbance in attention was reported as a TEAE by 2 subjects in the study (both in the mirogabalin 15‐mg BID cohort); however, this TEAE showed no correlation with mirogabalin dose (Table 1).

#### 3.4.3 Dizziness

In the 50‐ and 75‐mg cohorts of the single‐dose study, self‐reported dizziness, as measured by the VSS‐SF questionnaire, was apparent; values returned to predose levels by 24 hours postdose in the 50‐mg but not the 75‐mg cohort (Figure S1E). Similarly, dizziness was reported as an AE by more subjects in the 50‐mg (*n* = 3, 50.0%) and 75‐mg cohorts (*n* = 4, 66.7%) than in lower‐dose cohorts (*n *≤* *1 per cohort) (Table 1). In the multiple‐dose study, subjects receiving 15, 20, or 25 mg BID reported increases in dizziness from study day 3 (Figure S1F). At day 3, dizziness was reported as a TEAE for 5 subjects in the 15‐mg BID group, 1 subject in the 20‐mg BID group, and 2 subjects in the 25‐mg once‐daily (QD) to BID group (Table 1). The incidence of dizziness decreased after day 3. There was not a clear correlation between dizziness reported as a TEAE by the subject and VSS‐SF total scores.

#### 3.4.4 Ataxia

Subjects in the highest dose cohorts (50 and 75 mg) in the single‐ascending‐dose study had greater levels of ataxia as detected than those in other dose cohorts (Figure S1G). In these dose groups, TEAEs related to balance and gait were also reported.

In the multiple‐ascending‐dose study, ataxia increased in subjects receiving 15, 20, or 25 mg BID. This ataxia peaked between days 3 and 6 and returned to baseline thereafter; the highest increases in BARS scores were in the 20‐mg BID cohort (Figure S1H). A correlation between impaired balance or gait disturbance and BARS scores is suggested; however, mean BARS scores did not seem to detect symptoms with greater sensitivity than TEAE occurrence.

## 4 DISCUSSION

This article describes the first‐in‐human studies of mirogabalin, a preferentially selective ligand of the α_2_δ‐1 subunit of voltage‐dependent calcium channels, in healthy young and elderly subjects. Safety results from these 3 studies indicate that mirogabalin at doses ≤30 mg day^−1^ were well tolerated. Additionally, single doses of mirogabalin at 15 mg were well tolerated when administered in the fed and fasted states.

For all PD assessments, mirogabalin demonstrated dose‐dependent effects. In the single‐ascending‐dose study, mirogabalin was well tolerated over a dose range of 3‐30 mg. The subjects receiving the highest mirogabalin doses (50 and 75 mg) showed greater impairment as measured by PD assessments (increased dizziness, sedation, and ataxia, and worsened attention) and higher rates of TEAEs than subjects receiving 3‐30 mg. It was concluded that 50‐ and 75‐mg doses were above the maximally tolerated dose for mirogabalin. In the multiple‐ascending‐dose study, doses of up to 15 mg BID were well tolerated. Higher doses were associated with an increased incidence and/or severity of CNS‐related TEAEs (somnolence, dizziness, balance disorder, cognitive disorder, and visual impairment) and thus, were not considered well tolerated. Almost all DSST scores increased over time in the multiple‐ascending‐dose study, indicating an increase in correct responses. Hence, a learning curve during the study is evident, despite prestudy training to prevent this.

TEAEs of dizziness and somnolence were expected based on the mirogabalin mechanism of action. Therefore, it was not unexpected that, in both the single‐ and multiple‐ascending‐dose studies, dizziness and somnolence were among the most commonly reported TEAEs. However, with pregabalin treatment, subjects developed tolerance to these TEAEs.^15^ In the multiple‐ascending‐dose study, somnolence or dizziness resolved or improved within 4‐5 days, suggesting that tolerance developed to these CNS TEAEs. Asymptomatic elevation of hepatic transaminase levels was reported in 1 subject receiving mirogabalin 10 mg BID in the multiple‐ascending‐dose study. Although mild, these elevations were considered related to study treatment, and the subject was discontinued from the study.

Results of the single‐ and multiple‐ascending‐dose studies confirmed that mirogabalin is rapidly absorbed and rapidly eliminated with a dose‐proportional increase in exposure, but with no significant accumulation over 14 days of dosing. Urinary excretion data indicate that a large percentage of an orally administered dose is excreted renally, suggesting high oral bioavailability of >85%. The rate of renal clearance of mirogabalin is higher than the glomerular filtration rate, indicating possible active renal secretion of mirogabalin. The results of these studies support the Biopharmaceutical Classification System class determination for mirogabalin.

The findings of the food effect study demonstrated that, when mirogabalin was administered with a high‐fat meal, absorption was delayed, but overall exposure was not affected. The effect of food on drug absorption can be mediated via a number of mechanisms, including delayed gastric emptying, a change in gastrointestinal pH, and presence of high fat.^16^ Such effects can significantly alter the bioavailability of orally administered drugs, which can affect pharmacological response or drug safety. These observations are consistent with the high solubility and permeability characteristics of mirogabalin. The delayed absorption of mirogabalin when administered with a meal could be attributable to a food‐induced delay in gastric emptying, as observed with pregabalin.^14^

Based on these data, mirogabalin 15 mg BID was selected as the highest target dose for further clinical development. The extent of mirogabalin absorption is considered equivalent in the fed and fasted states and, because long‐term administration of mirogabalin is planned, delayed absorption was not considered a clinically relevant issue when mirogabalin is administered with food. Therefore, it is recommended that mirogabalin be taken irrespective of fed or fasted status in phase 3 trials. Phase 3 trials are underway to evaluate the efficacy and safety of mirogabalin in patients with DPNP (NCT02318706) and postherpetic neuralgia (NCT02318719).

## DISCLOSURES

H.Z. and J.M.‐H. are employees of Daiichi Sankyo, Inc. At the time the study was conducted, K.B., V.W., and V.D. were employees of Daiichi Sankyo and K.B. owned stock in the company. C.H., V.W., L.H., and S.O. have nothing to disclose.

## AUTHOR CONTRIBUTIONS

K.B., V.D., J.M., S.O., and H.Z. designed the study; K.B., J.M., S.O., C.H., L.H., V.W., V.D., and H.Z. contributed to data acquisition, analysis, or interpretation; and K.B., J.M., S.O., C.H., V.W., V.D., and H.Z. drafted the work or revised it critically. All authors revised and approved the final version of the manuscript and agreed to be accountable for all aspects of the work.
