# Supplementary material for: Tolerability, pharmacokinetics, and pharmacodynamics of mirogabalin in healthy subjects: Results from phase 1 studies
Source: Pharmacol Res Perspect. 2018 Aug 23;6(5):e00418. doi: 10.1002/prp2.418 (PMC6106189; doi:10.1002/prp2.418)
Supplement: Supplementary file 1 [file PRP2-6-e00418-s001.docx]

**Supplementary Data 1**

**INCLUSION AND EXCLUSION CRITERIA FOR THE SINGLE AND MULTIPLE ASCENDING-DOSE STUDIES AND THE FOOD EFFECT STUDY**

***Inclusion criteria***

- Women were required to be of nonchildbearing potential (either surgically sterile ≥6 months before dosing or naturally postmenopausal ≥24 consecutive months before dosing, with a follicle-stimulating hormone level at screening ≥40 mIU mL^–1^). In the single and multiple ascending-dose studies, a negative serum pregnancy test result was required at screening and within the 48 hours preceding dosing
- In the food effect study only, women of childbearing potential were recruited, provided they practiced an acceptable nonhormonal method of contraception for ≥3 months before study screening and agreed to use a condom with spermicide, in addition to their usual method, from screening through 30 days after their last dose of the study, or agreed to abstain from sexual activity for the study duration and for 30 days after the last dose of study drug
- Subjects were required to be in good health before enrollment (defined as absence of clinically significant deviation from normal, based on medical history, physical examination, laboratory reports and 12-lead electrocardiography
- Consumption of food or beverages containing alcohol was prohibited from 24 to 48 hours before check-in through discharge from the clinic
- In the multiple ascending-dose study only, consumption of grapefruit juice or grapefruit-containing products was prohibited within the 72 hours before check-in until discharge from the clinic

***Key exclusion criteria***

- History or presence of any clinically significant neurological or psychiatric disorders, or any other clinically significant disease
- Evidence of human immunodeficiency virus antibody, hepatitis B surface antigen, or hepatitis C antibody at screening
- Positive urine test results for drugs of abuse or alcohol at screening or check-in
- Taking medication known to interact with pregabalin or gabapentin or having a history of hypersensitivity to study drugs
- Enrollment in a previous dose cohort of the study or participation in another investigational new drug research study within the 30 days preceding day 1 of the current study
- In the multiple ascending-dose study, additional exclusion criteria included creatinine clearance <60 mL min^–1^; presence of any chronic pain condition (except mild osteoarthritis not necessitating long-term medication) or long-term use of analgesics/sedatives more than twice weekly; and needing medication that induced or inhibited CYP3A4 or CYP2D6 that could not be washed out for a minimum of 7 half-lives
- In the food effect study, the use of tobacco products or nicotine-containing products, including smoking cessation aids, within the 6 months preceding the first dose

**Supplementary Data 2**

**PHARMACODYNAMIC SCALES**

- Sedation was analyzed in both studies using the Line Analog Rating Scale (LARS) [1,2] and Profile of Mood States (POMS) [3].
  - For the LARS, subjects were asked to mark their present state along 10-cm lines labeled ‘drowsy’, ‘tired’, ‘alert’, and ‘energetic’, with the mid-point of each line representing their normal condition. To assess sedation, the individual distances (mm) for each line were measured and added to obtain the total score, with a higher total score indicating a greater degree of sedation
  - The POMS comprises 65 questions on how a subject was feeling at the time of the test, with responses ranging from 0 (not at all) to 4 (extremely); responses were totaled and converted to T scores (between 30 and 80). The analysis variables for the POMS were the T scores for tension-anxiety, depression-dejection, anger-hostility, vigor-activity, fatigue-inertia, and confusion-bewilderment, and the total mood disturbance score
- Attention was evaluated using the Digit Symbol Substitution Test (DSST; both studies) [4]
  - In the DSST, subjects must substitute figures for symbols. The total number of substitutions and the number of correct substitutions obtained within 90 seconds was recorded
- Dizziness was evaluated using the Vertigo Symptom Scale Short Form (VSS-SF) [5], a self-rated scale with 15 questions asking subjects how often in the past day they had experienced each vertigo symptom (vertigo-balance- or autonomic-anxiety-related)
- Ataxia was assessed by the Brief Ataxia Rating Scale [6] a 5-item neurological examination (gait, 0–8; knee-tibia test, 0–4; finger-to-nose test, 0–4; dysarthria, 0–4; and oculomotor abnormalities, 0–2), in which a score of 0 = normal for each item

**REFERENCES**

1 Gudgeon AC and Hindmarch I. Midazolam: Effects on psychomotor performance and subjective aspects of sleep and sedation in normal volunteers. Br J Clin Pharm 1983; 16(suppl 1): 121S–126S.

2 Hindmarch I, Trick L, and Ridout F. A double-blind, placebo- and positive-internal-controlled (alprazolam) investigation of the cognitive and psychomotor profile of pregabalin in healthy volunteers. Psychopharmacology (Berl) 2005; 183: 183(2): 133–143.

3 Pollock V, Cho DW, Reker D, and Volavka J. Profile of mood states: the factors and their psychological correlates. J Nerv Mental Dis 1979; 167(10): 612–614.

4 Hinton-Bayre A, Geffen G. Comparability, reliability, and practice effects on alternate forms of the digit symbol substitution and symbol digit modalities tests. Psychological Assessment 2005; 17(2): 237–241.

5 Kondo M, Kiyomizu K, Goto F, Kitahara T, Imai T, Hashimoto M, *et al*. Analysis of vestibular-balance symptoms according to symptom duration: dimensionality of the Vertigo Symptom Scale-short form. Health Qual Life Outcomes 2015; 13(1): 4.

6 Schmahmann JD, Gardner R, MacMore J, and Vangel MG. Development of a Brief Ataxia Rating Scale (BARS) based on a modified form of the ICARS. Mov Disord. 2009; 24(12): 1820–1828.

**FIGURE LEGEND**

**Figure S1.** Mean profile of pharmacodynamic scores. A) Sedation (LARS), single ascending-dose study; B) LARS, multiple ascending-dose study; C) Attention (DSST), single ascending-dose study; D) DSST, multiple ascending-dose study; E) Dizziness (VSS-SF), single ascending-dose study; F) VSS-SF, multiple ascending-dose study; G) Ataxia (BARS), single ascending-dose study; H) BARS, multiple ascending-dose study. For the multiple ascending dose graphs, the numbers in parenthesis on the time axis represent hours post-morning dose. The horizontal grey line in B, D, E, F, and H represents 0. BARS, Brief Ataxia Rating Scale; DSST, Digit Symbol Substitution Test; LARS, Line Analog Rating Scale; VSS-SF, Vertigo Symptom Scale Short Form.

**Table S1.** Baseline demographics

| **Single ascending-dose study** | | | | | | | | |
| --- | --- | --- | --- | --- | --- | --- | --- | --- |
|  | **Mirogabalin** | | | | | |  |  |
|  | **3 mg (*n* = 6)** | **5 mg (*n* = 6)** | **10 mg (*n* = 6)** | **30 mg (*n* = 6)** | **50 mg (*n* = 6)** | **75 mg (*n* = 6)** | **Placebo (*n* = 12)** | **Overall (*N* = 48)** |
| Withdrew from study, *n* (%) | 0 | 0 | 0 | 0 | 0 | 0 | 0 | 0 |
| Age, mean (range), y | 27.2 (19-32) | 31.7  (25-42) | 33.5 (27-43) | 29.3 (19-39) | 35.2 (23-44) | 32.3 (22-44) | 31.1 (21-38) | 31.4 (19-44) |
| Male, *n* (%) | 6 (100.0) | 6 (100.0) | 5 (83.3) | 6 (100.0) | 6 (100.0) | 4 (66.7) | 12 (100.0) | 45 (93.8) |
| BMI, mean (SD), kg/m^2^ | 26.5 (3.7) | 24.5 (1.9) | 25.7 (3.1) | 24.6 (3.5) | 25.6 (1.7) | 25.3 (2.8) | 23.7 (2.1) | 24.9 (2.7) |
| Race, *n* (%)  White  Black/African American  Asian  Other^a^ | 2 (33.3)  2 (33.3)  1 (16.7)  1 (16.7) | 3 (50.0)  2 (33.3)  0  1 (16.7) | 3 (50.0)  2 (33.3)  0  1 (16.7) | 4 (66.7)  2 (33.3)  0  0 | 4 (66.7)  2 (33.3)  0  0 | 4 (66.7)  1 (16.7)  0  1 (16.7) | 7 (58.3)  5 (41.7)  0  0 | 27 (56.3)  16 (33.3)  1 (2.1)  4 (8.3) |
| **Multiple ascending-dose study** | | | | | | | | |
| **Mirogabalin** | | | | | |  |  |  |
|  | **5 mg BID (*n* = 6)** | **10 mg BID (*n* = 6)** | **15 mg BID (*n* = 6)** | **20 mg BID (*n* = 6)** | **25 mg QD to BID  (*n* = 6)** | **Pregabalin 150 mg BID (*n* = 8)** | **Placebo**  **(*n* = 10)** | **Overall**  **(N = 48)** |
| Withdrew from study, *n* (%) | 0 | 1 (16.7)^b^ | 0 | 0 | 0 | 0 | 0 | 1 (2.1) |
| Age, mean (range), y  ≥65 years, *n* (%) | 64.8  (55-74)  3 (50.0) | 61.5  (56-68)  1 (16.7) | 59.3 (56-67)  1 (16.7) | 60.8 (56-72)  1 (16.7) | 59.3 (55-65)  1 (16.7) | 60.9 (55-73)  2 (25.0) | 62.5 (56-67)  3 (30.0) | 61.4 (55-74) 12 (25.0) |
| Male, n (%) | 5 (83.3) | 2 (33.3) | 4 (66.7) | 5 (83.3) | 3 (50.0) | 4 (50.0) | 8 (80.0) | 31 (64.6) |
| BMI, mean (SD), kg/m^2^ | 25.5 (2.4) | 24.6 (2.1) | 24.9 (4.1) | 26.9 (3.1) | 25.7 (2.2) | 26.9 (2.6) | 27.3 (1.9) | 26.1 (2.7) |
| Race, *n* (%)  White  Black/African American  Asian  Other^a^ | 6 (100.0)  0  0  0 | 5 (83.3)  1 (16.7)  0  0 | 6 (100.0)  0  0  0 | 5 (83.3)  0  0  1 (16.7) | 5 (83.3)  0  1 (16.7)  0 | 7 (87.5)  0  0  1 (12.5) | 10 (100.0)  0  0  0 | 44 (91.7)  1 (2.1)  1 (2.1)  2 (4.2) |
| **Food effect study** | | | | | | | | |
|  |  | | | | | | | **Mirogabalin**  **15 mg (N = 30)** |
| Withdrew from study, *n* (%) |  |  |  |  |  |  |  | 0 |
| Age, mean (range), y |  |  |  |  |  |  |  | 35.9 (18-58) |
| Male, *n* (%) |  |  |  |  |  |  |  | 19 (63.3) |
| BMI, mean (SD), kg/m^2^ |  |  |  |  |  |  |  | 25.5 (3.0) |
| Race, *n* (%)  White  Black/African American  Asian  Other^c^ |  |  |  |  |  |  |  | 18 (60.0)  10 (33.3)  0  2 (6.7) |

BID, twice daily; BMI, body mass index; QD, once daily; SD, standard deviation.
^a^Other consisted of American Indian or Alaskan native.

^b^The single subject who discontinued the study early had a mild treatment-emergent adverse event of elevated hepatic transaminase level.

^c^Other consisted of American Indian, Alaskan native and Hawaiian or Pacific Islander.

**Figure S1.**

**Sedation – Line Analog Rating Scale**

**Single ascending-dose study Multiple ascending-dose study**

**
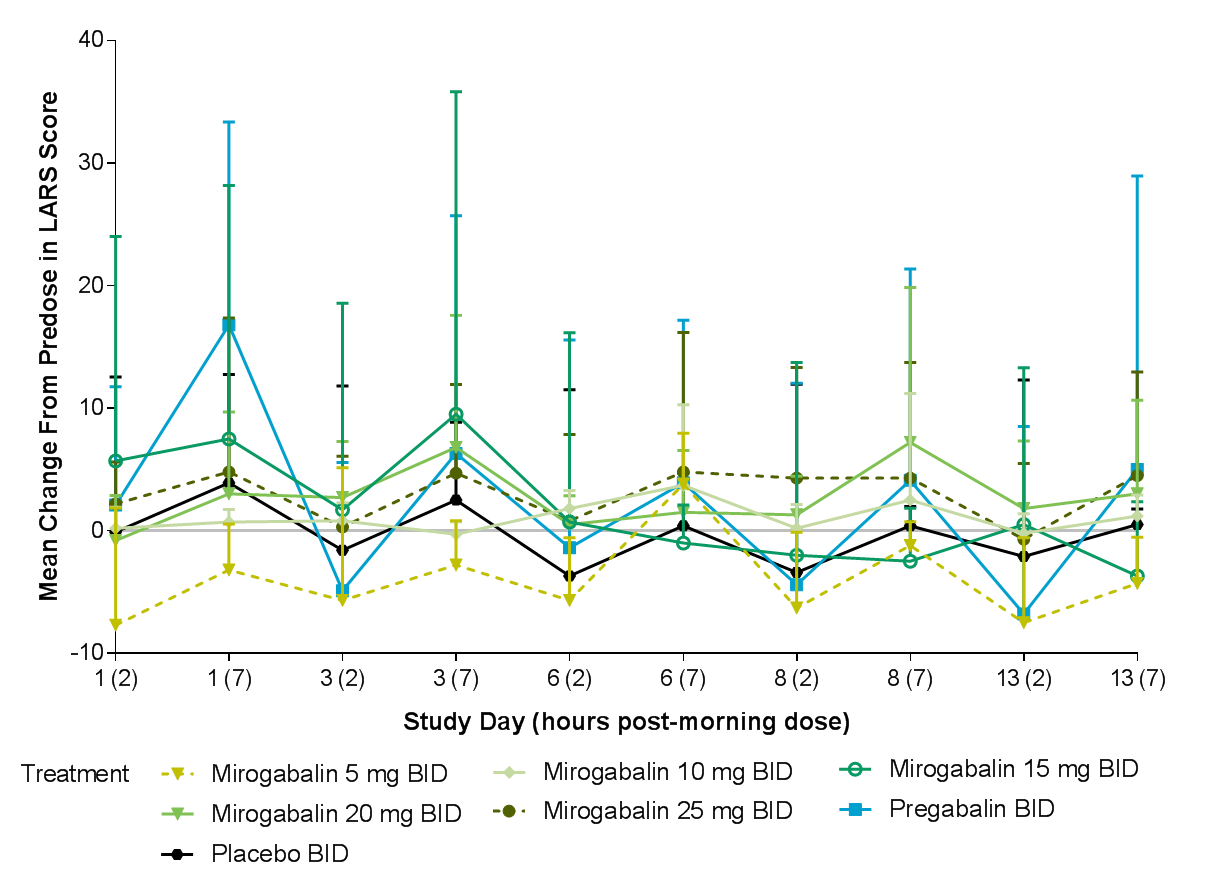

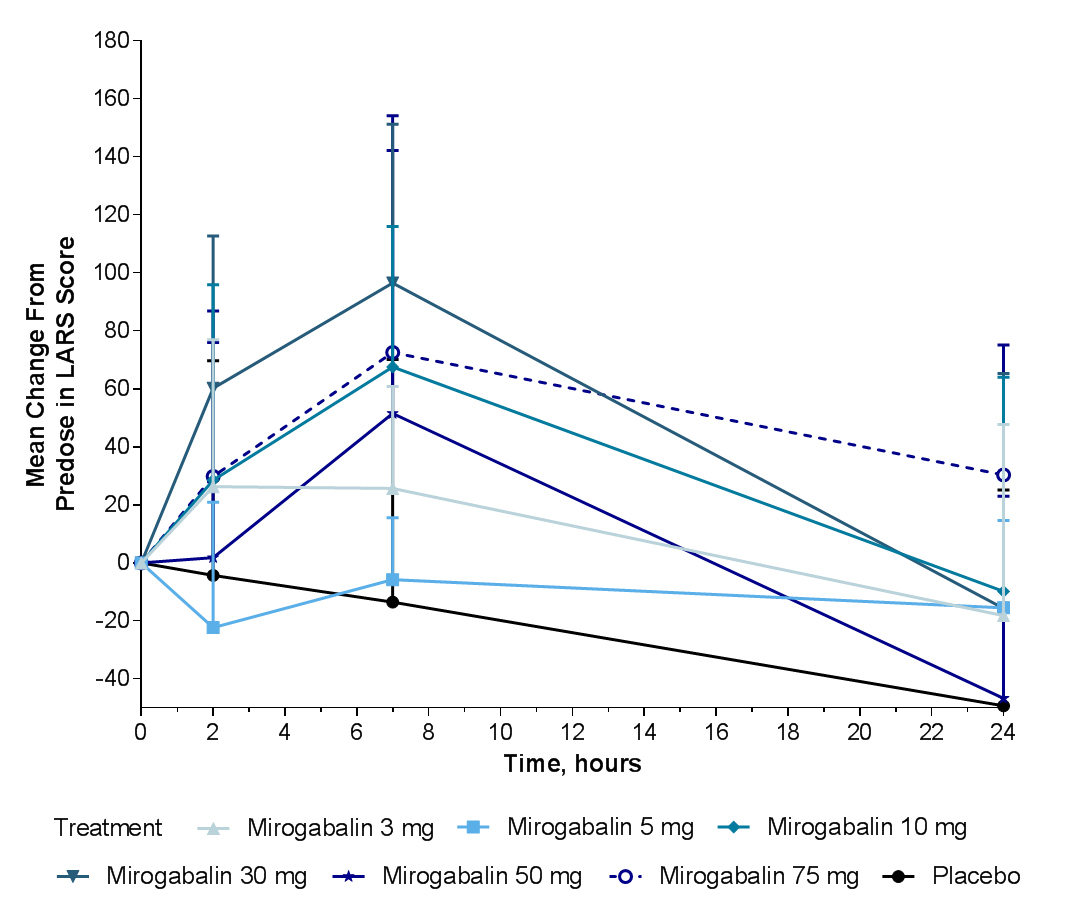
A B**

**Attention – Digit Symbol Substitution Test**

**Single ascending-dose study Multiple ascending-dose study**

**
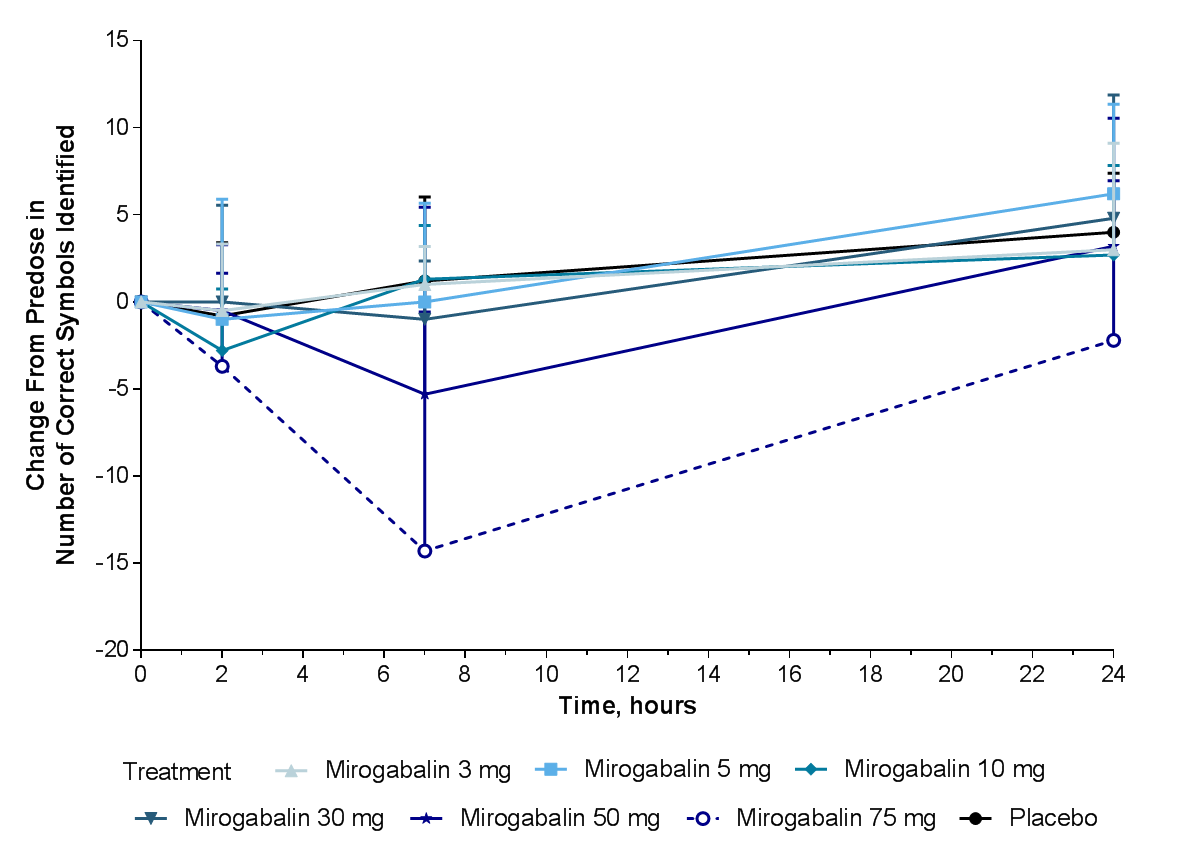

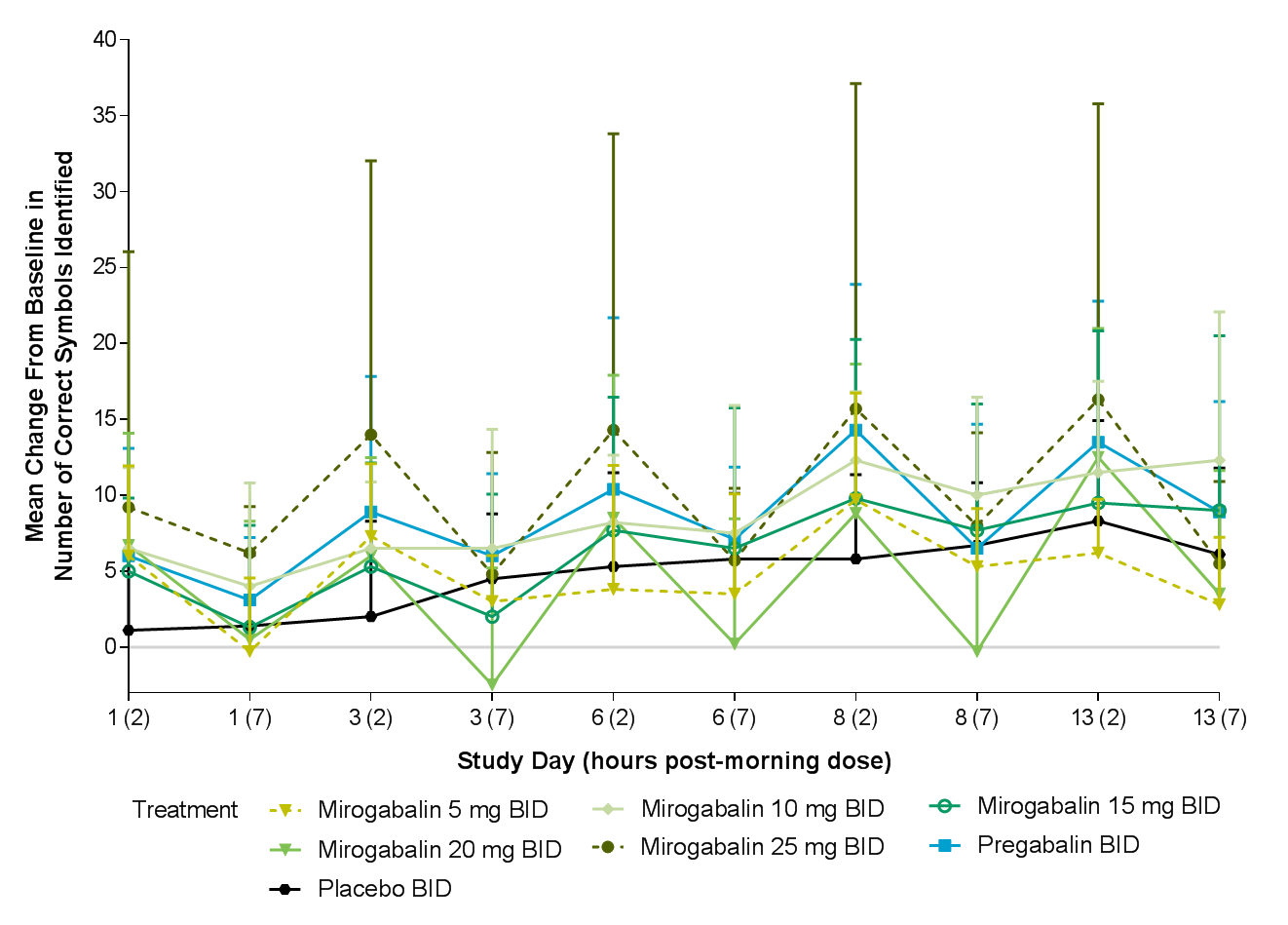
C D**

**Dizziness – Vertigo Symptom Scale-Short Form**

**Single-ascending dose study Multiple-ascending dose study**

**
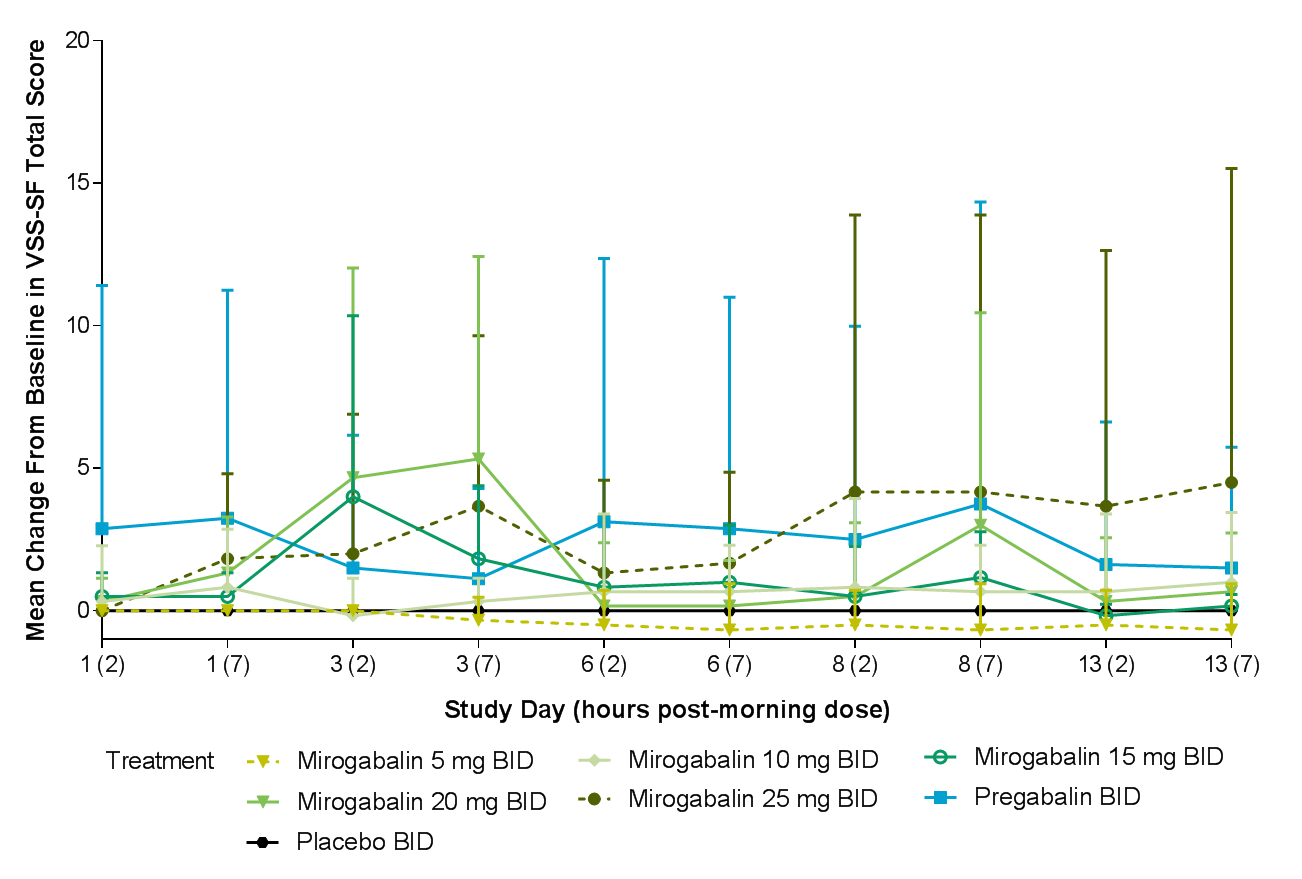

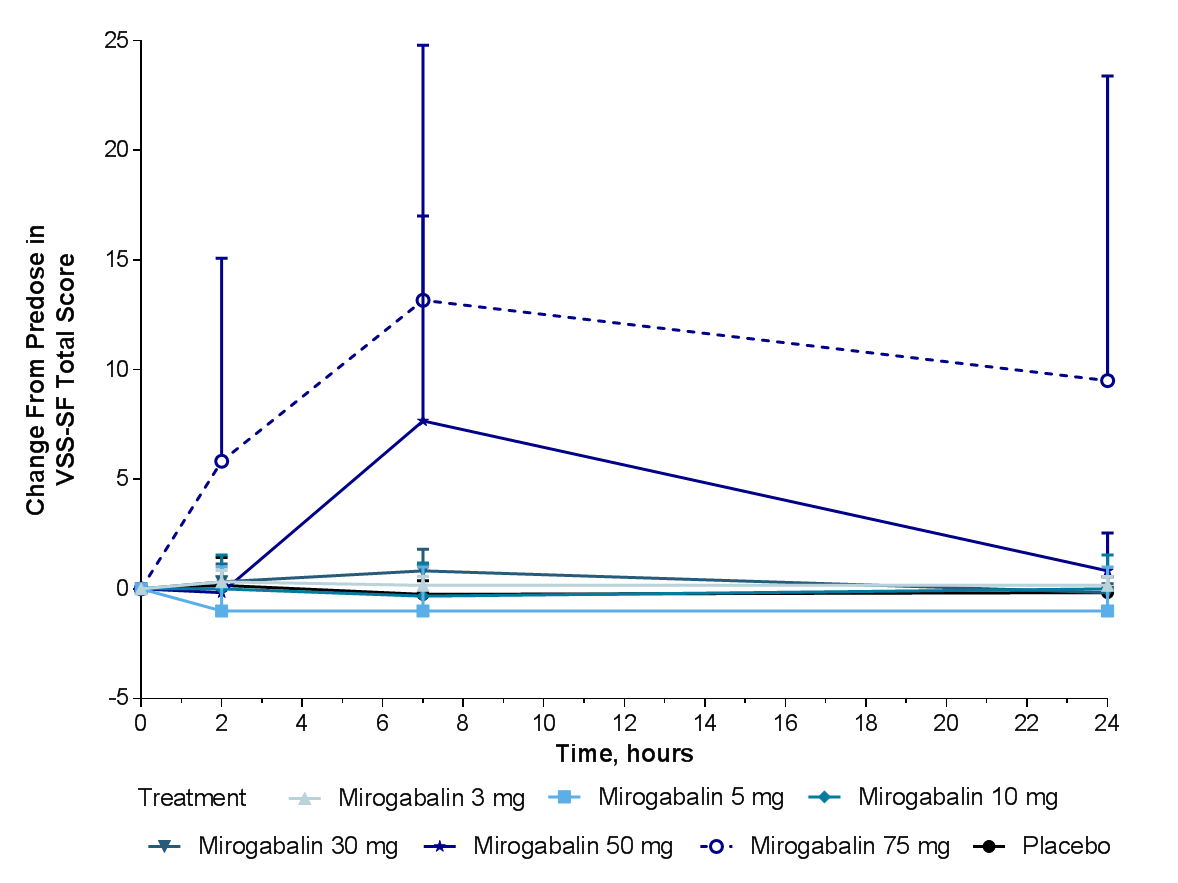
E F**

**Ataxia – Brief Ataxia Rating Scale**

**Single-ascending dose study Multiple-ascending dose study**

**
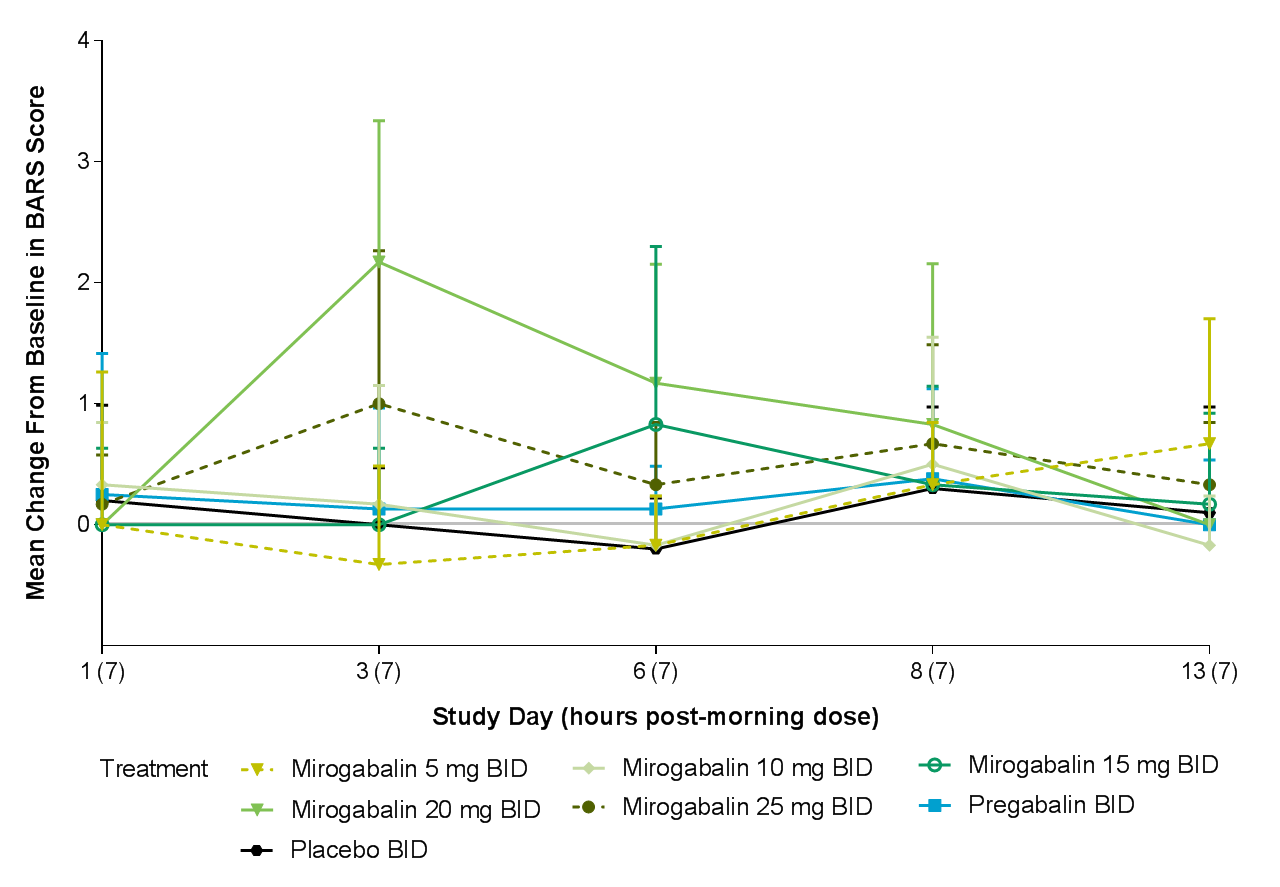

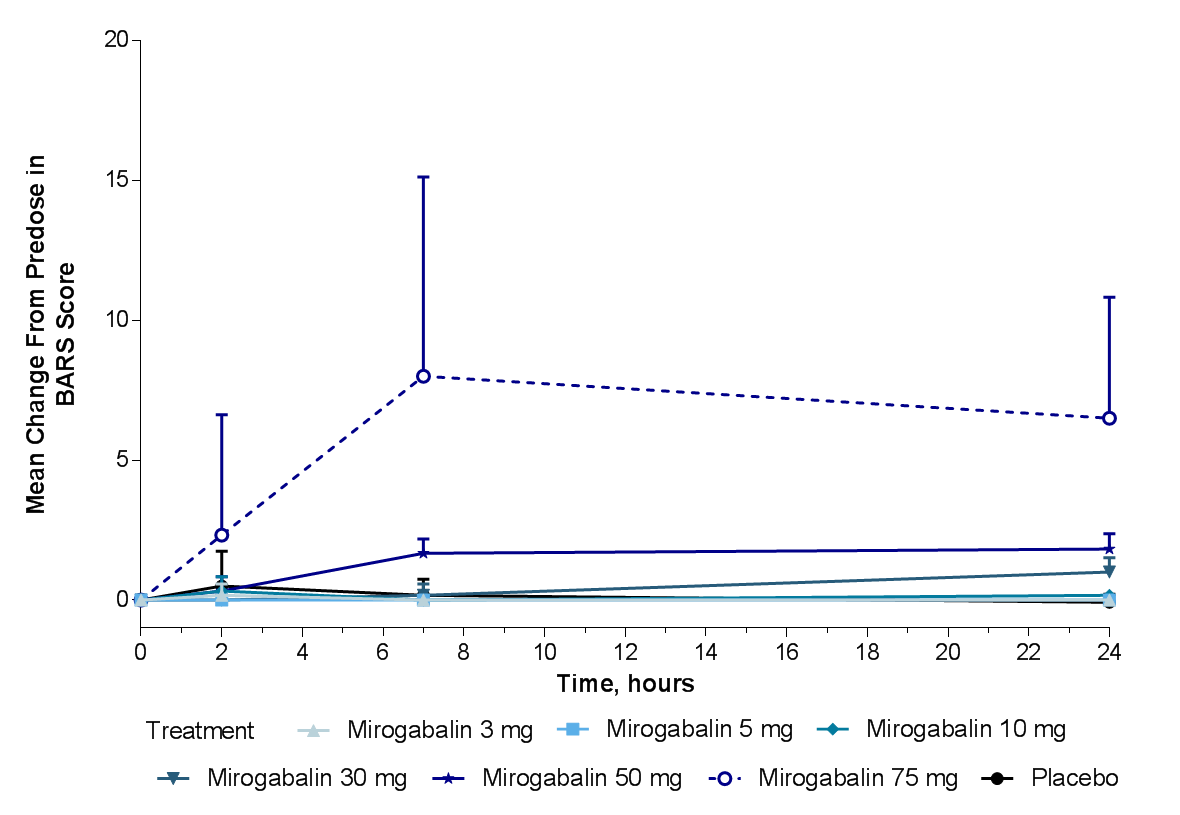
G H**
